# Molecular docking and dynamics simulation analysis of the human FXIIa with compounds from the Mcule database

**DOI:** 10.6026/97320630019160

**Published:** 2023-02-28

**Authors:** Hasanain Abdulhameed Odhar, Ahmed Fadhil Hashim, Salam Waheed Ahjel, Suhad Sami Humadi

**Affiliations:** 1 Department of pharmacy, Al-Zahrawi University College, Karbala, Iraq

**Keywords:** human plasma β-FXIIa, docking, molecular dynamics simulation, structure-based virtual screening, Mcule database

## Abstract

The human factor XIIa is a serine protease enzyme that is implicated in the pathological thrombosis. This coagulation factor represents an interesting molecular target to design safer antithrombotic agents without adversely influencing physiological hemostasis. Therefore, it is of interest to virtually screen the human factor XIIa crystal with millions of compounds in Mcule database in order to identify potential inhibitors. For this purpose, both molecular docking and dynamics simulation were employed to identify potential hits. Also, various predictive approaches were utilized to estimate chemical, pharmacokinetics and toxicological features for the top hits. As such, we report here that compound 4 (1-(4-benzylpiperazin-1-yl)-2-[5-(3,5-dimethylpyrazol-1-yl)-1,2,3, 4-tetrazol-2-yl]ethanone) may be a potential ligand against the human factor XIIa for further consideration in the design and development of novel antithrombotic agents.

## Background:

**Figure 1:**
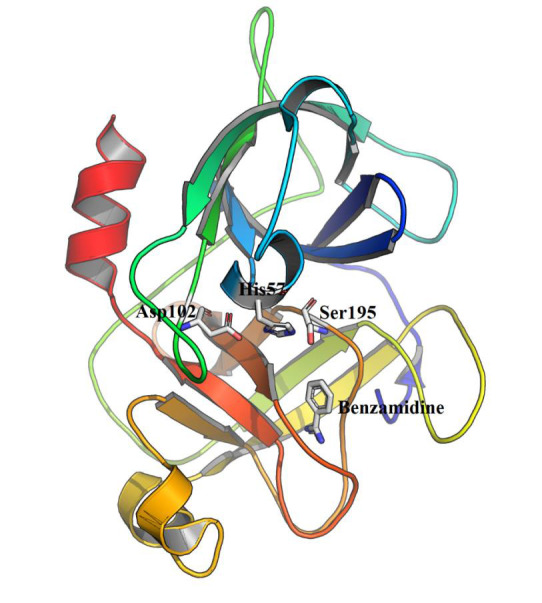
A three-dimensional cartoon illustration for the human plasma β-FXIIa in complex with the non-covalent inhibitor benzamidine (PBD: 6B74). Residues of the catalytic triad are represented as labelled sticks.

The human factor XIIa (FXIIa) is a serine protease enzyme that is essential for the launch of the intrinsic coagulation pathway in a contact activation reaction involving both plasma prekallikrein (PPK) and high molecular weight kininogen (HMWK) [1]. In this activation reaction, the plasma FXII zymogen will undergo a proteolytic transformation into the active protease FXIIa. This proteolytic conversion process occurs on a negatively charged surface where a small quantity of activated FXII is generated [2]. In the same time, HMWK attached to the same surface will deliver PPK to FXIIa for activation. Then, this generated active plasma kallikrein (PK) will reciprocally activate extra quantity of FXIIa [3]. And during the later steps of intrinsic coagulation cascade, FXIIa will cleave its substrate FXI to produce FXIa, which in turn converts FIX to FIXa [4, 5]. Finally, this cascade of events will drive the generation of thrombin and fibrin formation. It has been demonstrated that FXIIa activation of FXI is not substantial for hemostasis therefore it is believed that the deficiency of FXII is not usually associated with excessive bleeding whether in humans or animals [3, 4, 5, 6]. In addition to the procoagulant activity, the FXIIa mediated contact system has a proinflammatory effect through the kallikrein-kinin pathway, which can release bradykinin from HMWK by PK [7]. As such, FXIIa represents an attractive molecular target to design and introduce new drug candidates that can hinder pathological thrombus formation and without adversely influencing physiological hemostasis. The use of available antithrombotic drugs like warfarin, heparin and antiplatelet agents can be related to a high risk of severe bleeding because these drugs can target components of the blood clotting cascade like thrombin, FVIIa, FIXa, FXa, FXIa [3]. Structure-based virtual screening is a computational approach that can identify potential hits against a molecular target with a known three-dimensional structure [8]. In this in-silico study, both molecular docking and dynamics simulation were employed to carry out structure-based virtual screening against human plasma β-FXIIa crystal in order to identify potential inhibitors. For this purpose, we have used the human plasma β-FXIIa complexed with benzamidine as a target to perform structure-based virtual screening [3]. A cartoon representation for the three-dimensional structure of the human plasma β-FXIIa co-crystalized with benzamidine can be seen in Figure 1.

## Methodology:

## Setting up a plan for virtual screening study:

**Figure 2:**
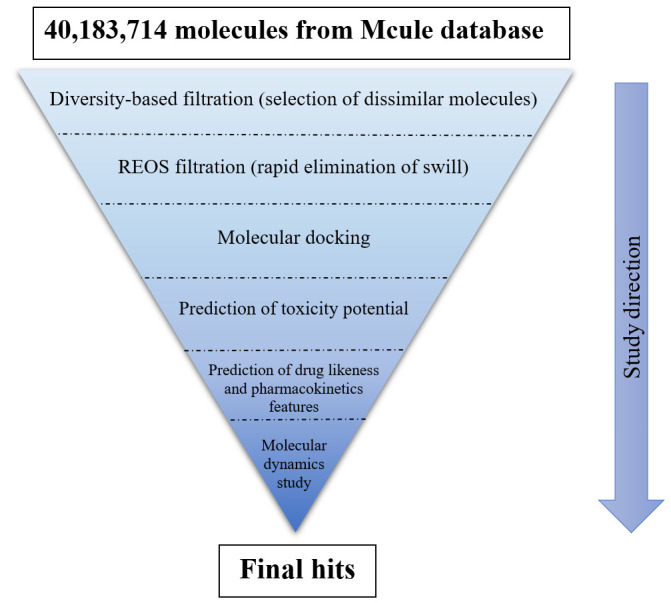
A schematic representation for the main steps of virtual screening study.

The major steps for this in-silico screening study can be summarized in Figure 2. As seen in this figure, more than 40 million compounds were filtered through different screening steps. Concisely, the database of these chemical compounds was first subjected to diversity-based filtration to select the most dissimilar molecules. Then, REOS (rapid elimination of swill) filter was used to remove any frequent and non-selective compounds in the database. After that, molecular docking was applied to virtually assess binding of these compounds to the active site of the human plasma β-FXIIa. Moreover, prediction of toxicity potential, drug-likeness and pharmacokinetics were utilized to further refine the screening of these compounds. Finally, two rounds of molecular dynamics (MD) simulation for 20 and 50 nanoseconds were applied to the filtered compounds.

## Structure-based virtual screening:

For this stage, we have employed the structure-based virtual screening pipeline available through the online drug discovery website Mcule.com [9]. The methodology and options of this structure-based virtual screening are identical to what we had used in our previously published studies [10, 11, 12, 13]. In this stage, the full Mcule database with more than 40 million compounds was used for this screening study. At first, the diversity-based filter was applied in order to choose the most dissimilar compounds. Then, the REOS (rapid elimination of swill) filter was utilized to eliminate non-selective hitters. After that, molecular docking was employed to assess the binding of these filtered compounds against the human plasma β-FXIIa. Both AutoDock tools and AutoDock Vina, embedded in Mcule.com website, were used to carry out molecular docking [14,15]. For the docking step, the human plasma β-FXIIa crystal (PBD: 6B74) was used as a target [3]. The employed docking coordinates were X: 62, Y: -1, Z: 15 while the dimensions for the used grid box were 22*22*22 Angstrom. To validate this docking protocol, the non-covalent inhibitor benzamidine was redocked to the plasma β-FXIIa crystal. Then, the Root Mean Square Deviation (RMSD) was calculated by comparing the conformations of the docked and the co-crystalized benzamidine [16]. Finally, the hits in the docking output were ranked based on their least binding energy and only the top ten compounds were selected for further validation. Both PyMOL v2.4.1 and Discovery Studio Visualizer v21.1.0 were used to inspect the docking orientation of the least binding energy pose for each compound against the plasma β-FXIIa crystal [17, 18]. And by examination of docking interaction, we have excluded any compound with unfavorable interaction against target active site.

## Prediction of toxicity potential, drug-likeness score and pharmacokinetics properties:

Then, the refined compounds were subjected to prediction of median lethal dose (LD50) by using ProTox-II webserver [19]. Also, the AMES mutagenic potential for each hit was anticipated by using pkCSM sever [20]. Regarding pharmacokinetics features for these hits, both pkCSM and SwissADME servers were utilized to predict volume of distribution and water solubility [20, 21]. And lastly, the drug-likeness score was predicted for each hit by using the molsoft website [22].

## Molecular dynamics (MD) simulation study:

The aim of this MD simulation study is to calculate ligand proximity to the plasma β-FXIIa active site and to estimate Molecular Mechanics Poisson-Boltzmann Surface Area (MM-PBSA) binding energy for the refined hits during simulation period. To accomplish this objective, The YASARA Dynamics v20.12.24 was used to carry out two rounds of MD simulation for 20 and 50 nanoseconds [23]. Initially, the MD simulation was executed for each refined hit for 20 nanoseconds only. Then, only those hits with an average RMSD of ligand movement that is less than 4 Angstrom were subjected to another round of MD simulation for 50 nanoseconds. The detailed procedure used for this MD simulation is like to what we had employed in our previously published articles [11, 12, 13]. In summary, 0.9% of NaCl was used for this MD simulation study and an excess concentration of either Na+or Cl ‾ was added to ensure neutralization of ligand-target complex. Both steepest descent and simulated annealing minimizations were employed to abolish clashes likelihood during simulation. throughout this simulation, the following force fields were employed: AMBER14 for the solute, TIP3P for water molecules, AM1BCC and GAFF2 for the ligands [24, 25, 26]. GraphPad Prism v8.0.2 was used to plot RMSD values of ligand movement during the simulation period of 50 nanoseconds [27]. For the estimation of MM-PBSA binding energy, the YASARA Dynamics utilizes AMBER14 force field where the more positive binding energy indicates better interactions between ligand and target [28]. The YASARA Dynamics software employed the following equation to estimate MM-PBSA binding energy:

Binding Energy = EpotRecept + EsolvRecept + EpotLigand + EsolvLigand - EpotComplex - EsolvComplex

## Results and Discussion:

**Figure 3:**
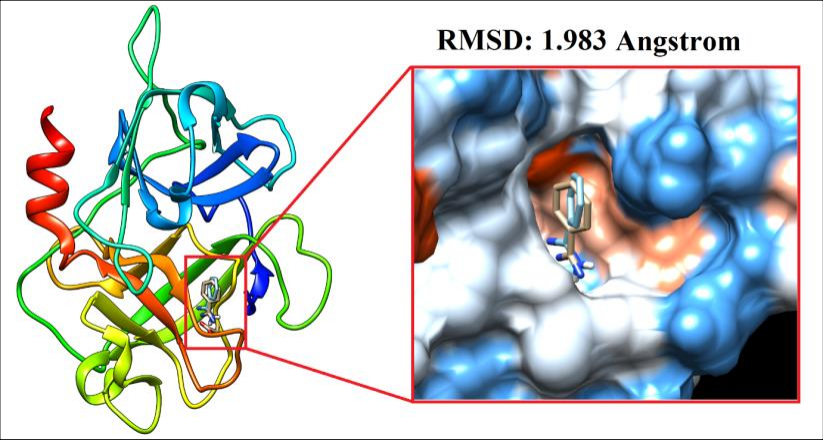
Redocking of the non-covalent inhibitor benzamidine into the β-FXIIa.

**Figure 4:**
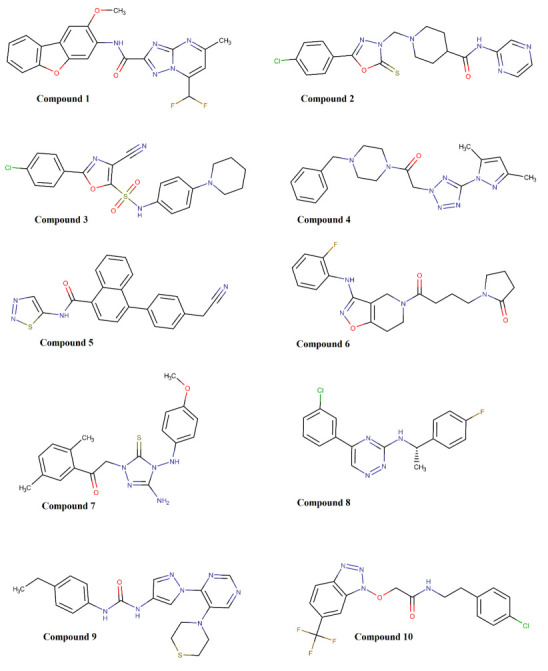
Chemical structures for the top ten hits that were virtually screened against the plasma β-FXIIa crystal.

**Figure 5:**
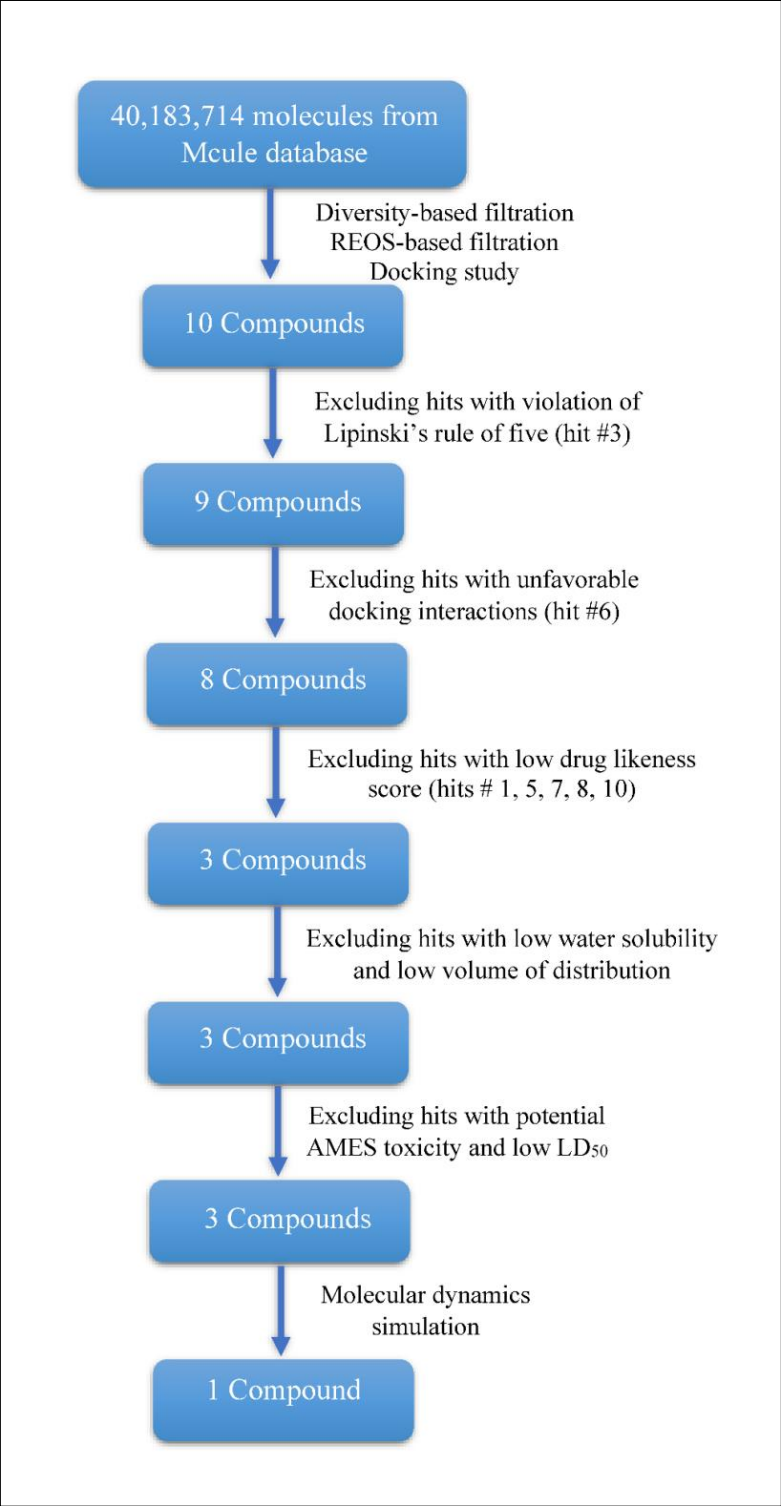
A concise overview for the stages of this structure-based virtual screening together with results for each filtration stage.

At first, the accuracy of docking protocol was validated by using pose selection (redocking) approach. In this approach, the co-crystalized ligand is removed from target crystal and redocked into the same active site. Then, RMSD value for the difference between docked conformation and native conformation for that ligand is calculated. It is believed that RMSD value for the conformations difference between 1.5 and 2 Angstrom refers to a reliable docking protocol [29]. In this regard, our redocking result for the co-crystalized benzamidine indicates that the followed docking protocol is genuine. The RMSD value for the conformations difference between docked and co-crystalized ligand was 1.983 Å as seen in Figure 3. According to the chemical characteristics presented in Table 1 for the top 10 hits screened against the plasma β-FXIIa, almost all of these hits are predicted to have good oral bioavailability [30,31]. The only exception is hit number 3 with a logarithm of octanol/ water partition coefficient (Log P) that is greater than 5. In addition, the chemical structure of these top hits can be seen in Figure 4. Then, a tabular summary can be seen in Table 2 for the docking results, pharmacokinetics features and toxicity potential of these top hits. As noted in this table, the only compound that showed unfavorable interactions with target crystal in docking study is hit number 6. And based on the prediction of drug-likeness score for each hit in Table 2, we can notice that four hits have a high score and these are compounds 2, 4, 6 and 9. While prediction of water solubility indicated that hits 3 and 5 may have poor solubility. On the other hand, volume of distribution (VD) anticipation referred to the possibility that compounds 1, 3, 5, 7, 8 and 10 may have low VD values. Finally, evaluation of toxicity potential prediction in Table 2 pointed out to the mutagenic probability of both compounds 5 and 7. Also, both compounds 1 and 6 have a relatively low median lethal dose (LD50) as predicted in Table 2. Based on prediction results in Table 1 and Table 2, it is evident that compounds 2, 4 and 9 may represent potential candidates that can be further evaluated in molecular dynamics (MD) study. The overall results of this in-silico methodology can be summarized in Figure 5.

**Figure 6:**
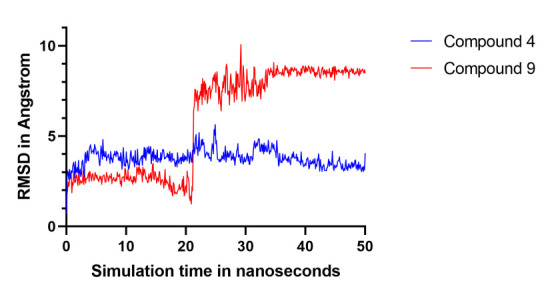
Ligand movement RMSD for compounds 4 and 9 against the human plasma β-FXIIa throughout simulation interval. This plot was generated by superposing ligand-target complex on its reference structure.

**Figure 7:**
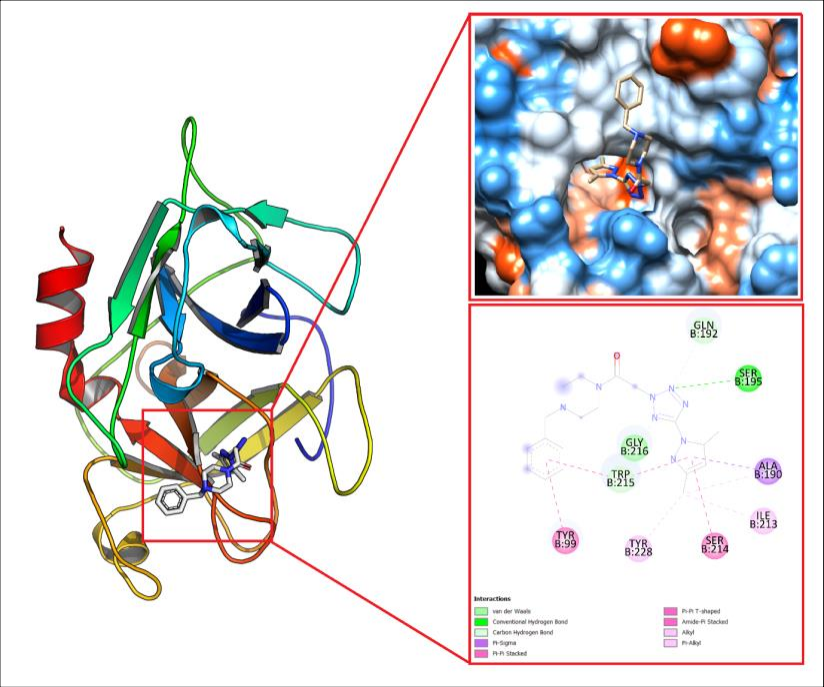
A cartoon representation for the docking of compound 4 into the human plasma β-FXIIa crystal.

As molecular dynamics (MD) simulation is considered a time-consuming process that requires extensive computational resources. Therefore, compounds 2, 4 and 9 were subjected at first to MD simulation for only 20 nanoseconds. It is well-known that ligand proximity to the target active site during simulation usually refers to stronger ligand binding to its target. This ligand proximity to target active site can be estimated by calculating ligand movement Root Mean Square Deviation (RMSD) throughout simulation [32,33]. As such, we have evaluated the mean RMSD value for ligand movement from target active site during simulation and subsequently eliminated those hits with mean RMSD value greater than 4 Angstrom. A tabular summary for MD simulation study can be seen in Table 3 for these three hits. According to Table 3, it is evident that compound 2 has a mean RMSD value of 6.84 Å during ligand movement analysis. As a result, only compounds 4 and 9 were then subjected to an extended MD simulation for 50 nanoseconds period. Analysis of this extended simulation indicated that compound 4 was able to maintain a close proximity to target crystal with a mean RMSD value of 3.78 Å. While the mean RMSD of ligand movement for compound 9 was 5.81 Å throughout 50 nanoseconds. These results of ligand movement are in agreement with the calculated average MM-PBSA binding energy for compounds 4 and 9 which was 9.82 and -17.2 Kcal/mol respectively. As mentioned in YASARA Dynamics guideline, the more positive MM-PBSA binding energy points out to better interactions between ligand and target crystal [28]. Based on these findings in Table 3, it seems that compound 4 may be a better ligand to the human plasma β-FXIIa as compared to compound 9. This is because compound 4 was able to maintain a lower RMSD value of ligand movement throughout simulation. Also, the average MM-PBSA binding energy for compound 4 was more positive unlike compound 9. The detailed analysis of ligand movement RMSD for both compounds 4 and 9 throughout 50 nanoseconds period of simulation can be seen in Figure 6. Lastly, a cartoon illustration for the docking of compound 4 into the human plasma β-FXIIa crystal can be seen in Figure 7. It is evident from this figure that compound 4 is involved in hydrogen bonding with Serine 195, a key residue of the catalytic triad.

## Conclusion:

Here, we report that compound 4 (1-(4-benzylpiperazin-1-yl)-2- [5-(3,5-dimethylpyrazol- 1-yl)-1,2,3,4-tetrazol-2-yl] ethanone) may represents a novel ligand for the human plasma β-FXIIa crystal. Prediction analysis carried out in this in-silico study showed that compound 4 obeys Lipinski's rule of five for oral activity. It also has a good docking orientation, a high drug-likeness score and acceptable pharmacokinetics and safety profiles. Finally, analysis of ligand movement and binding energy during molecular dynamics simulation indicated that compound 4 may be a potential ligand for β-FXIIa crystal; however these computational results must be validated both in vitro and in vivo.

## Figures and Tables

**Table 1:** Chemical characteristics for the top hits that were generated by virtual screening of Mcule database library against the crystal of human plasma beta-factor XIIa (β-FXIIa). These best compounds were ranked based on their minimum binding energy to the β-FXIIa crystal

**Compound No**	**Molecular Formula**	**M.W. (g/ mol)**	**Log P**	**TPSA (Å^2^)**	**H-bond acceptors**	**H-bond donors**
1	C21H15F2N5O3	423.37	4.6	94.55	8	1
2	C19H19ClN6O2S	430.91	3.64	121.17	8	1
3	C21H19ClN4O3S	442.92	5.88	107.61	7	1
4	C19H24N8O	380.45	0.7	84.97	9	0
5	C21H14N4OS	370.43	4.75	106.91	5	1
6	C20H23FN4O3	386.42	2.79	78.68	7	1
7	C19H21N5O2S	383.47	4.03	119.19	7	2
8	C17H14ClFN4	328.77	4.58	50.7	4	1
9	C20H23N7OS	409.51	3.63	113.27	8	2
10	C17H14ClF3N4O2	398.77	3.28	69.04	6	1
M.W.: molecular weight; Log P: logarithm of partition coefficient; TPSA: topological polar surface area.

**Table 2:** A summary for docking results, predicted pharmacokinetic characteristics and toxicity potential for the top hits generated by virtual screening of Mcule library against β-FXIIa crystal. These chemical candidates were ranked according to their minimum docking energy to β-FXIIa crystal.

**No**	**Docking results**		**Drug-likeness score**		**Pharmacokinetics characteristics**	**Toxicity potential**	
	**Binding energy (Kcal/ mol)**	**Unfavorable interactions**		**Water solubility (mg/ ml)**	**VDss (L/Kg)**	**AMES toxicity**	**LD50 (mg/ Kg)**
1	-9.3	No	0.15	8.09E-04	0.7	No	300
				Moderate			
2	-9	No	2.15	8.80E-03	1.38	No	1000
				Moderate			
3	-8.7	No	-0.14	1.46E-04	0.65	No	1600
				Poor			
4	-8.7	No	0.92	1.01E-01	1.15	No	489
				Soluble			
5	-8.4	No	0.17	2.99E-04	0.76	Yes	1000
				Poor			
6	-8.4	Yes	0.92	5.82E-01	1.6	No	235
				Soluble			
7	-8.3	No	-0.28	6.68E-04	0.52	Yes	1800
				Moderate			
8	-8.3	No	0.24	9.07E-03	0.64	No	750
				Moderate			
9	-8.3	No	0.99	1.52E-02	1.05	No	800
				Moderate			
10	-8.3	No	0.12	1.28E-03	0.38	No	800
				Moderate			
VDss: steady state volume of distribution; LD50: median lethal dose.

**Table 3:** A summary for molecular dynamics (MD) simulation for the filtered top hits

**Hit no.**	**MD simulation for 20 nano seconds**			**MD simulation for 50 nano seconds**			
	**Ligand movement RMSD (Å)**			**Ligand movement RMSD (Å)**			**Average MM-PBSA binding energy (Kcal/ mol)**
	**Mean**	**Minimum**	**Maximum**	**Mean**	**Minimum**	**Maximum**	
2	6.84	0.74	12.67	-	-	-	-
4	3.63	0.76	4.9	3.78	0.76	5.63	9.82
9	2.59	0.72	3.48	5.81	0.72	10.05	-17.2
MD: Molecular dynamics; MM-PBSA: Molecular Mechanics Poisson-Boltzmann Surface Area; RMSD: Root-Mean-Square Deviation; Å Angstrom.
